# Pathologic response with neoadjuvant chemotherapy and stereotactic body radiotherapy for borderline resectable and locally-advanced pancreatic cancer

**DOI:** 10.1186/1748-717X-8-254

**Published:** 2013-10-31

**Authors:** Malolan S Rajagopalan, Dwight E Heron, Rodney E Wegner, Herbert J Zeh, Nathan Bahary, Alyssa M Krasinskas, Barry Lembersky, Randall Brand, A James Moser, Annette E Quinn, Steven A Burton

**Affiliations:** 1 Department of Radiation Oncology, University of Pittsburgh Cancer Institute, Pittsburgh, PA, USA; 2 Department of Surgical Oncology, University of Pittsburgh Cancer Institute, Pittsburgh, PA, USA; 3 Department of Medical Oncology, University of Pittsburgh Cancer Institute, Pittsburgh, PA, USA; 4 Department of Pathology, University of Pittsburgh Medical Center, Pittsburgh, PA, USA; 5 Department of Gastroenterology, University of Pittsburgh Medical Center, Pittsburgh, PA, USA; 6 Department of Surgery, Beth Israel Deaconess Medical Center, Boston, MA, USA; 7 Current address: Delta Medix Center for Comprehensive Cancer Care, Scranton, PA, USA

**Keywords:** Pancreatic cancer, Stereotactic body radiotherapy, SBRT, Neoadjuvant, Pathologic response

## Abstract

**Background:**

Neoadjuvant stereotactic body radiotherapy (SBRT) has potential applicability in the management of borderline resectable and locally-advanced pancreatic adenocarcinoma. In this series, we report the pathologic outcomes in the subset of patients who underwent surgery after neoadjuvant SBRT.

**Methods:**

Patients with borderline resectable or locally-advanced pancreatic adenocarcinoma who were treated with SBRT followed by resection were included. Chemotherapy was to the discretion of the medical oncologist and preceded SBRT for most patients.

**Results:**

Twelve patients met inclusion criteria. Most (92%) received neoadjuvant chemotherapy, and gemcitabine/capecitabine was most frequently utilized (n = 7). Most were treated with fractionated SBRT to 36 Gy/3 fractions (n = 7) and the remainder with single fraction to 24 Gy (n = 5). No grade 3+ acute toxicities attributable to SBRT were found. Two patients developed post-surgical vascular complications and one died secondary to this. The mean time to surgery after SBRT was 3.3 months. An R0 resection was performed in 92% of patients (n = 11/12). In 25% (n = 3/12) of patients, a complete pathologic response was achieved, and an additional 16.7% (n = 2/12) demonstrated <10% viable tumor cells. Kaplan-Meier estimated median progression free survival is 27.4 months. Overall survival is 92%, 64% and 51% at 1-, 2-, and 3-years.

**Conclusions:**

This study reports the pathologic response in patients treated with neoadjuvant chemotherapy and SBRT for borderline resectable and locally-advanced pancreatic cancer. In our experience, 92% achieved an R0 resection and 41.7% of patients demonstrated either complete or extensive pathologic response to treatment. The results of a phase II study of this novel approach will be forthcoming.

## Background

Pancreatic cancer is the fourth leading cause of death from cancer in both men and women in the United States [1]. It is a highly aggressive entity with approximately 40% presenting as locally-advanced but unresectable disease and an additional 40% presenting with metastatic disease [2]. Despite intensive efforts with chemotherapy and radiation therapy, surgical resection remains the only treatment option associated with long-term survival [3].

Chemotherapy and radiation therapy have been explored in the adjuvant setting. The GITSG trial was the only major clinical trial to demonstrate a survival benefit for adjuvant chemoradiation [4]. However, this study is routinely criticized for its small size, antiquated techniques and split-course radiation delivery. Furthermore, when attempted to be replicated by EORTC 40891, no benefit to adjuvant chemoradiation was demonstrated. Based upon this study and ESPAC-1, which does have a number of flaws with design but found a deleterious effect of adjuvant chemoradiation, the use of adjuvant radiation has fallen out of favor in Europe and is controversial in North America [5,6]. Meanwhile, a number of studies including CONKO-001 and RTOG 9704 began to shed light that gemcitabine-based adjuvant chemotherapy may be more efficacious than previous regimens [7,8]. However, it must be noted that the recent update to ESPAC-3 found that adjuvant gemcitabine may be equivalent to adjuvant 5FU [9].

Neoadjuvant therapy has a number of potential benefits compared to adjuvant therapy. It may, in theory, eradicate micrometastatic disease while potentially downsizing the primary lesion to facilitate a margin negative (R0) resection. Many patients may never recover sufficiently to tolerate adjuvant therapy. Finally, patients can be selected for surgery and those who develop metastatic disease during the course of neoadjuvant therapy can be spared the morbidity and mortality risks associated with a pancreaticoduodenectomy. Neoadjuvant therapy was studied at MD Anderson Cancer Center in a retrospective series of over 300 patients. This series lent evidence that induction chemotherapy followed by concurrent chemo-radiation for unresectable cancer improved progression-free survival and median survival when compared to patients who underwent chemoradiation alone [10].

Stereotactic Body Radiotherapy (SBRT) is particularly promising for pancreatic cancer. SBRT enables the delivery of high doses to a small target in few fractions. By completing radiotherapy in the span of one or two weeks, the patient is potentially able to benefit from full doses of chemotherapy without interruption, less toxicity, and the ability to proceed to surgery faster. In this study, we retrospectively analyzed patients with locally advanced or borderline resectable pancreatic cancer who underwent neoadjuvant therapy with SBRT and completed surgical resection.

## Methods

### Patient population

Patients with biopsy-proven borderline resectable or locally-advanced pancreatic adenocarcinoma who were treated with neoadjuvant SBRT from 2008–2011 and completed surgical resection were included in this retrospective analysis. Definitions for borderline resectability and locally advanced disease were based on radiographic criteria defined by Varadhachary [11]. All patients were seen, evaluated, and followed by a pancreatico-biliary surgeon, medical oncologist and radiation oncologist in the Pancreas Multidisciplinary Clinic at the University of Pittsburgh Cancer Institute. This study was conducted under a formal Institutional Review Board approved protocol.

### Chemotherapy

Most patients in this series received neoadjuvant chemotherapy. The choice of chemotherapy was to the discretion of the medical oncologist. Chemotherapy preceded SBRT whenever possible.

### Radiation therapy

For stereotactic localization, fiducial markers were required prior to SBRT. Two to three fiducial markers were placed in or around the tumor. All patients underwent a 4D-CT treatment simulation with a custom-made relocatable Alpha Cradle® immobilization system (Smithers Medical Products, North Canton, OH). Target and critical structures (liver, small bowel, stomach, kidneys, and spinal cord) were contoured. The GTV was defined as the tumor visible on the CT scan, and in those with N1 disease, the nodes were not included in the target. The GTV was expanded by 2 mm to form the PTV.

The radiosurgical plan was devised to typically deliver a dose of 24 Gy in a single fraction or 36 Gy in 3 fractions. Patients treated during or before 2010 were treated with a single fraction. The multidisciplinary group chose to fractionate SBRT starting in 2011 to reduce the potential for toxicity. Recommended normal tissue constraints guidelines for three fractions were as follows: spinal cord <18 Gy, stomach <30 Gy, small bowel <25 Gy, liver 15 Gy to <700 cm^3^, kidney 15 Gy to <1/3 total volume. For single fraction treatment, the small bowel maximum point dose was 18.5 Gy. Plans were devised such that the prescription dose was the isodose line encompassing >95% of the PTV. No more than 20% of the PTV was to receive a dose >110% of the prescription dose. No more than 2% of the PTV was to receive <93% of the prescription dose. Patients were treated on any of three radiosurgical platforms including the Accuray CyberKnife® (Accuray Inc., Sunnyvale, CA) or Varian Trilogy® or TrueBeam® (Varian Medical Systems Inc., Palo Alto, CA).

All patients underwent motion-compensated treatment when required as determined by motion of the target. On the CyberKnife® platform, the Synchrony™ respiratory tracking system was utilized. For those treated on Trilogy® or TrueBeam®, the Real-time Position Management™ Respiratory Gating System (Varian Medical Systems, Inc. Palo Alto, CA) was utilized when required.

### Surgery

Follow-up CT imaging was obtained in all patients 10–12 weeks after the completion of SBRT as was the standard protocol at our institution. If there was no evidence of distant metastasis and the malignancy appeared technically resectable, surgical exploration and curative resection was recommended. Surgery was performed by experienced pancreatico-biliary surgeons. Patients underwent either classical pancreaticoduodenectomy or pylorus preserving pancreaticoduodenectomy for head tumors and distal pancreatectomy for body/tail lesions. When there was involvement of the celiac or hepatic artery, an Appleby procedure may have been performed. When required, vein reconstruction was also performed.

### Pathology

All surgical pathology specimens were analyzed at the same lab in the University of Pittsburgh Cancer Institute. Negative margins were scored if there was at least a 1 mm margin of benign tissue. All pathologic specimens were re-reviewed and scored by an expert pathologist (AK). Pathologic response after neoadjuvant therapy was scored using the Evans’ criteria as detailed in Table 1[12].

**Table 1:** **Evans**’ **criteria for pathologic response following neoadjuvant therapy**

**Grade**	**Tumor regression**
**I**	<10% to no tumor cells destroyed
**II**	**IIa**: 10-50% of tumor cells destroyed
	**IIb**: 50-90% of tumor cells destroyed
**III**	>90% of tumor cells destroyed
	**IIIM**: sizable pools of cellular mucin
**IV**	No viable tumor cells
	**IVM**: Acellular pools of mucin

### Statistical methods

All figures are reported as mean ± standard deviation except when otherwise indicated. Kaplan-Meier survival analysis was used to estimate overall survival (OS) and progression free survival (PFS). Survival rates were calculated from the date of biopsy. Acute toxicities were defined as those occurring within 90 days of SBRT and late toxicities were defined as those occurring thereafter. SPSS® software version 18.0 (IBM Corp., Armonk, NY) was used for statistical analysis.

## Results

### Patient characteristics

One-hundred and five patients were treated at our institution with SBRT for pancreatic cancer for primary or definitive intent. Of these, twelve patients underwent surgical resection after SBRT and thus met criteria for inclusion in this retrospective series. The median age was 68 (range: 82–41 years) and 41.7% were male. All patients were considered to have locally advanced (n = 5/12) or borderline resectable (n = 7/12) disease as determined by experienced pancreatic surgeons. Patients were evenly divided between T3 (50%) and T4 (50%) stage. The majority of patients were N0, but 41.7% had N1 disease.

### Chemotherapy

Most patients (91.7%) had neoadjuvant chemotherapy following diagnosis and preceding SBRT. One patient did not have neoadjuvant chemotherapy due to recent bone marrow transplant for multiple myeloma. In this case, single-agent gemcitabine was deferred to the adjuvant setting. Among those who received neoadjuvant chemotherapy, it was gemcitabine-based in the vast majority (90.9%) and most received gemcitabine /capecitabine (n = 7/11). Other neoadjuvant chemotherapy regimens included gemcitabine/erlotinib, gemcitabine alone, FOLFIRINOX and gemcitabine/Abaxane® followed by FOLFIRINOX. Following surgery, the majority of patients (75%) did receive additional chemotherapy in the adjuvant setting.

### Stereotactic body radiotherapy

**Figure 1:**
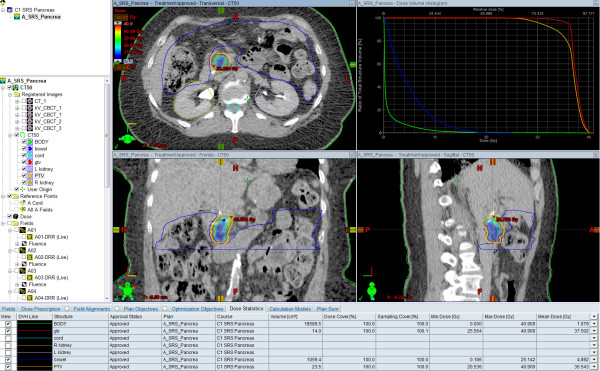
Treatment plan for a patient treated with SBRT to a dose of 36 Gy / 3 fractions following neoadjuvant chemotherapy with gemcitabine/capecitabine.

SBRT was delivered at a single site by experienced radiation oncologists. Treatment was delivered on either the CyberKnife® platform for 33% of the patients and the remaining patients were treated on the Varian Trilogy™ or TrueBeam™ platforms. Patients treated from 2008 – 2010 received a single fraction dose of 24 Gy (n = 5/12) while those treated in 2011 typically received a hypofractionated course of 36 Gy in 3 fractions (n = 6/12) and one received 30 Gy in 3 fractions (Figure 1). The mean target volume was 16.6 cm^3^ (range: 5.1 – 44.6 cm^3^). Intensity modulated radiosurgery plans typically involved 11–13 fields while those on the CyberKnife® platform utilized 150–170 beams. Maximum spinal cord doses were 1.56 Gy (range: 1.0 – 1.8 Gy) for single fraction plans and 6.6 Gy (range: 1.3 – 10.2 Gy) for hypofractionated plans. Maximum bowel doses were 13.7 Gy (range: 11.3 – 16.1 Gy) and 21.6 Gy (16.8 – 25.1 Gy) for single and multi-fraction plans respectively. There were no acute grade 3 or higher toxicities directly attributable to SBRT.

### Surgery

The mean time to surgery following SBRT was 3.3 months (range: 1.5 – 6.6 months). Vein reconstruction was performed in 25% of cases. Postoperative complications included pseudoaneruysm, bradycardia, infection and bleeding. There was one patient who suffered both post-operative infection and bleeding in the postoperative period and eventually succumbed to this. This was felt to be a technical complication related to the surgery and narrow vasculature and not related to SBRT. However, two other patients developed pseudoaneurysms which are thought to be related to SBRT. One patient, who underwent surgery 6.6 months following SBRT, developed a pseudoaneurysm of the gastroduodenal artery in the absence of a pancreatic leak and required coiling. She recovered well from the procedure. Another patient, who underwent surgery 4.5 months after SBRT, developed a pseudoanerusym of the splenic artery stump with gastrointestinal bleeding. This patient required multiple procedures approximately 6.5 months following surgery, and eventually died of complications related to this. After multidisciplinary review, the group believes that these two vascular complications are related to a long interval between SBRT and definitive surgical management.

### Pathology

Final pathology revealed that an R0 resection with at least a 1 mm margin was achieved in the vast majority of patients (91.7%). There was only one patient who had a positive margin and this patient had borderline resectable disease at diagnosis. All patients who underwent a vein reconstruction achieved an R0 resection.

**Figure 2:**
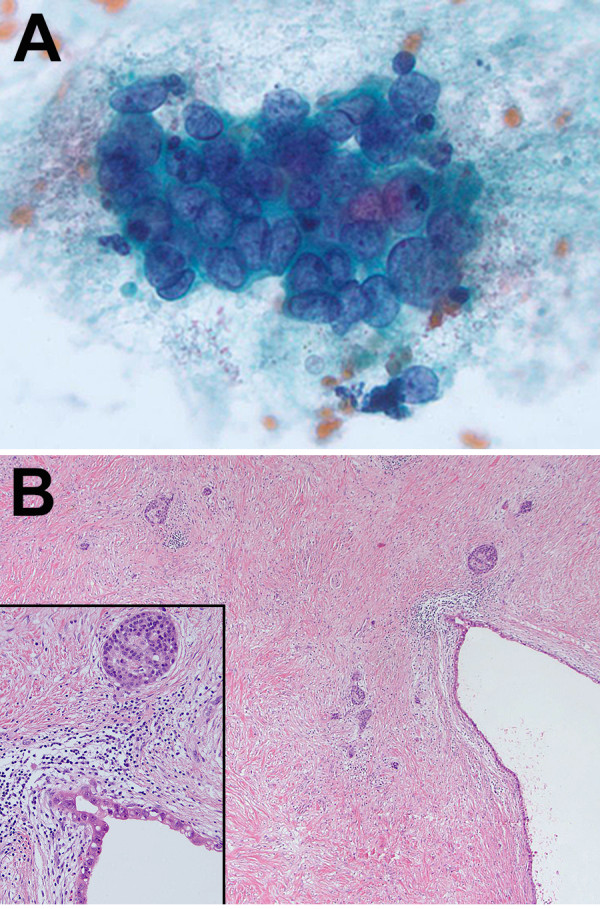
**The same patient had a complete pathologic response following neoadjuvant chemotherapy and SBRT.** Panel **A** is from the pre-treatment fine needle aspiration and shows adenocarcinoma. Panel **B** demonstrates extensive fibrosis in the surgical resection post-treatment with no evidence of invasive adenocarcinoma. The small nests of cells that are present within the dense fibrosis are all islets (*=cystically dilated duct lined by PanIN-3). Inset shows higher magnification of the boxed area, including one islet and PanIN-3.

Three patients (25%) achieved a complete pathologic response (pCR) to neoadjuvant therapy (Figure 2). These patients were treated with SBRT to 36 Gy in three fractions. Two patients (16.7%) had >90% of tumor cells destroyed and an additional two (16.7%) had > 50% tumor cell destruction when scored by Evans’ criteria (Table 2). Thus 41.7% of patients had at least 90% tumor cell destruction in response to neoadjuvant therapy and a total of 58.3% had at least 50% tumor cell destruction.

**Table 2:** Pathologic response following neoadjuvant therapy

	**Neoadjuvant chemotherapy**	**Neoadjuvant SBRT (dose / fractions)**	**Pathologic response (Evans’ criteria)**
**01**	Gem./Cape.	36 Gy / 3	IV
**02**	Gem./Cape.	36 Gy / 3	IV
**03**	FOLFIRINOX, Gem./Abraxane	36 Gy / 3	IV
**04**	Gem./Erlotinib	24 Gy / 1	III
**05**	Gem./Cape.	24 Gy / 1	III
**06**	Gem.	24 Gy / 1	IIb
**07**	FOLFIRINOX	36 Gy / 3	IIb
**08**	N/A	36 Gy / 3	IIa
**09**	Gem./Cape.	36 Gy / 3	IIa
**10**	Gem./Cape.	24 Gy / 1	IIa
**11**	Gem./Cape.	24 Gy / 1	IIa
**12**	Gem./Cape.	30 Gy /3	IIa

25% of patients achieved a complete pathologic (pCR) response to neoadjuvant therapy (Evans’ grade: IV), and an additional 16.7% of patients had >90% tumor cell destruction (Evans’ grade: III). In total, 58.3% of patients had at least 50% of tumor cell destruction (Evans’ Grade: IIb – IV). For those achieving pCR, SBRT dose was 36 Gy in 3 fractions and neoadjuvant chemotherapy was gemcitabine-based. [*Abbreviations*: Gem Gemcitabine, Cape Capecitabine].

### Survival

**Figure 3:**
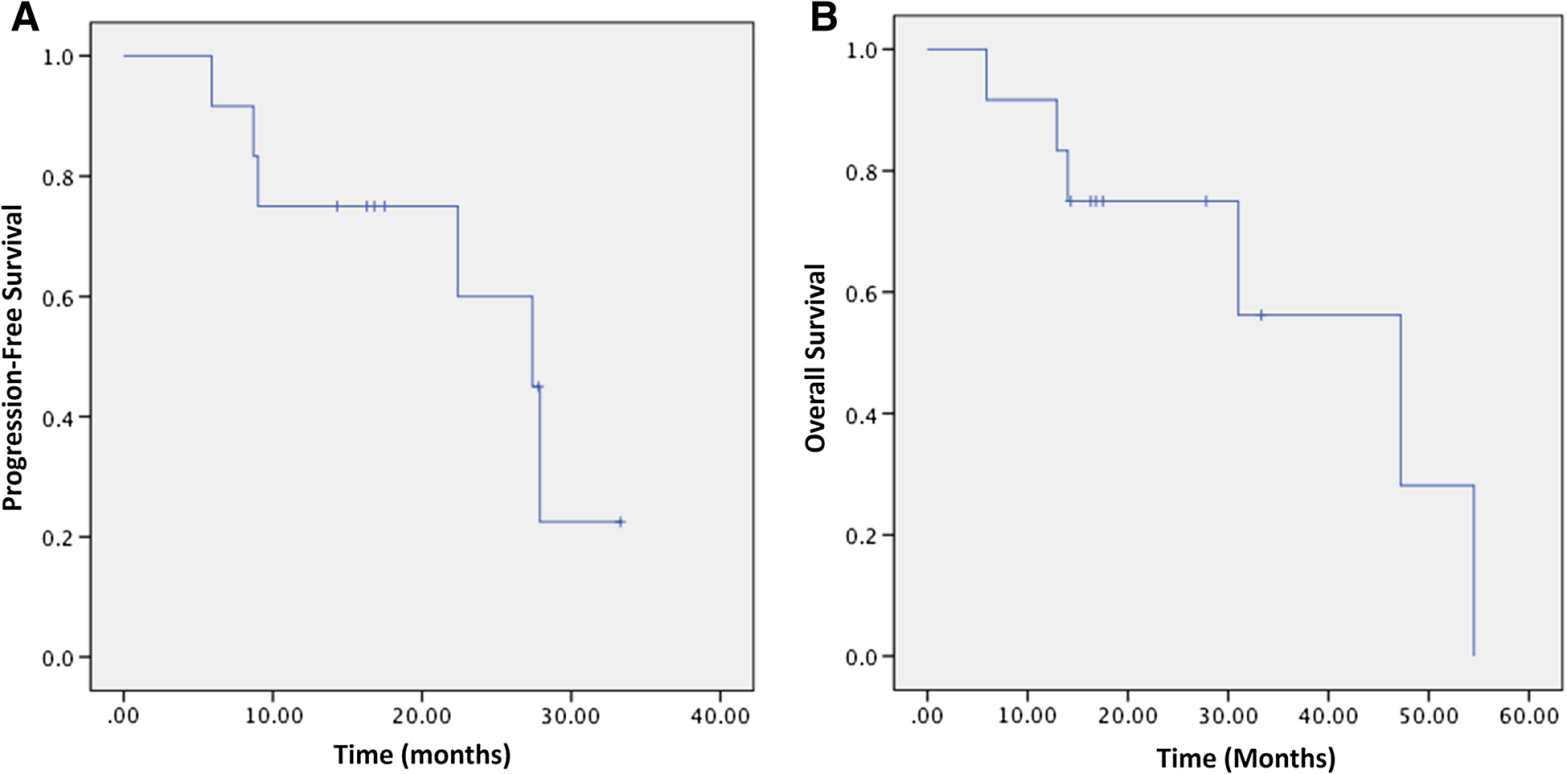
**Kaplan-Meier curves for progression-free survival (Panel A) and overall survival (Panel B).** The median progression free survival was 27.4 months. The median overall survival is 47.2 months. Overall survival is 92%, 64% and 51% at 1-, 2- and 3-years respectively.

Survival data is available with a median follow-up of 16.6 months (range: 10.5 – 54.5 months). Kaplan-Meier estimated 1-, 2-, and 3-year OS is 92%, 64% and 51% respectively (Figure 3). Kaplan-Meier estimated median survival is 47.2 months and median PFS is 27.4 months. Due to the small numbers in this study, subset analyses correlating time to surgery and pathologic response and pCR or R0 resection to outcome were not significant.

## Discussion

Herein we present pathologic outcomes in a series of patients with locally advanced and borderline resectable pancreatic cancer who underwent neoadjuvant SBRT. We found that in this cohort, the pCR rate was 25% and an additional 16.7% had an extensive response to therapy with >90% tumor cell destruction. The estimated 1-, 2-, and 3-year OS is 92%, 64% and 51%.

Most of the published studies analyzing SBRT have been performed in locally advanced, unresectable disease. Memorial Sloan Kettering has reported outcomes for patients treated in a phase II study with sequential gemcitabine and SBRT approach (with chemotherapy preceding and following SBRT) [13]. SBRT was delivered as a single fraction of 25 Gy using the Varian Trilogy® platform. Toxicities reported included duodenal perforation requiring surgery (5%). Median survival was 11.8 months and 1-year survival was 50%. The same group has previously reported a similar series of patients receiving gemcitabine and a single fraction of 25 Gy of SBRT delivered on the CyberKnife® platform [14]. Similar outcomes were reported with 11.4 months median survival and 1-year survival of 50% was noted. Acute toxicities included gastric outlet obstruction (6.3%). Late gastrointestinal toxicities included duodenal perforation and stricture (12.5%). Our institution has also reported a series of SBRT for locally advanced, unresectable disease [15]. The vast majority of patients were treated with a single fraction of 24 Gy. The rate of acute grade 3 or higher toxicities was 1.4%. Median survival in this series was 10.3 months and 1-year OS was 41%. A Harvard series assessing gemcitabine and fractionated SBRT (24 – 36 Gy) in the same population revealed a 14% grade 3 toxicity rate with MS of 14.3 months [16]. Finally, Stanford has reported on 77 patients with unresectable disease receiving single fraction of 25 Gy and demonstrated a 1-year rate of freedom from local progression of 84%, 1-year PFS of 9%, and 1-year OS from SBRT 21%. They report a 9% late grade 3 or higher toxicity [17].

Toxicity with pancreatic SBRT is a particular concern and has come under scrutiny. The Danish group has reported on a phase II study of SBRT for locally advanced pancreatic cancer in which a central dose of 45 Gy in three fractions was delivered [18]. They found that there was a significant deterioration in performance status, pain and nausea associated with SBRT within 2 weeks, but most improved by 3 months. They found that 18% (n = 4/22) suffered severe gastrointestinal ulceration or mucositis and one had perforation of the stomach. The Mayo Clinic undertook a dosimetric analysis of 47 patients undergoing abdominal SBRT to targets in proximity to hollow viscous organs [19]. A variety of dose and fractionations were used, but the most common was 50 Gy/5 fractions. There were no acute grade 3 or higher toxicities. Five late gastrointestinal toxicities were found, but no dose correlation could be ascertained. This report, taken in conjunction with the number of other reports detailing pancreatic SBRT, demonstrates the technique is feasible and can be performed with an acceptable toxicity profile. Acute toxicities are infrequent and in this report no acute grade 3 or higher toxicities were seen. It is hypothesized that the late gastrointestinal toxicity rate attributable to SBRT may be less frequent in our approach since the distal stomach and duodenum, which can stricture, ulcerate or perforate, is resected shortly after SBRT. There were two patients who developed pseudoaneurysm in the post-operative period in the absence of pancreatic leak. Thus we suspect this is related to ischemic injury secondary to SBRT. In both these patients, the interval from SBRT to surgery was greater than 4 months. We now believe that a shorter interval may reduce the potential for toxicity. We have since modified our protocol to obtain follow-up imaging 4 weeks following SBRT with the goal of performing surgery with a shorter interval from SBRT.

In the preoperative setting, two reports describe patients treated with SBRT who underwent resection. An Italian group studied patients with initially unresectable pancreatic cancer who underwent gemcitabine followed by SBRT and resection when feasible. Of the 23 patients who were enrolled on protocol, only two underwent resection. Both had an R0 resection but both had positive peri-pancreatic lymph nodes. One was found to a pCR in the primary site and the other had a partial response. Moffitt has reported on a large series of patients undergoing induction chemotherapy and SBRT for locally advanced and borderline resectable disease [20]. Of those that completed surgery, 97% had an R0 resection and the pCR rate was 9.4%.

There are a number of studies reporting pathologic outcomes following neoadjuvant chemotherapy with or without conventionally fractionated radiation therapy. A French group explored perioperative chemo-radiation and found a pCR rate of 7.5% (n = 3/40) [21]. MD Anderson Cancer Center has reported a phase II approach with neoadjuvant chemotherapy and radiation therapy and found a 2.7% pCR rate [22]. In a review of the experience at MD Anderson from 1995 – 2010, only 2.5% of patients who underwent neoadjuvant therapy and pancreatectomy achieved pCR. The authors concluded that, while rare, pCR was associated with extended survival [23].

There are limitations with our study as there are with all retrospective reports. Of the 105 patients treated with SBRT, only the twelve who completed both neoadjuvant therapy and surgery were included in the study and for survival analysis. Furthermore, the neoadjuvant approach, by design, selects for patients who do not develop distant metastases or extensive local progression that preclude use of SBRT. Thus, survival estimates reported in this series are expected to be higher than other reports. Finally, since most patients received neoadjuvant chemotherapy and SBRT, it is unknown if one of these modalities was the primary effector of the pathologic response.

Despite these limitations, however, this approach and these results are certainly promising. We believe an aggressive, tri-modality approach provides patients the greatest chance for a durable response and potential cure. Surgery has always remained the mainstay and those who are able to have it performed certainly benefit [3]. Chemotherapy has also proven benefit in a number of trials and is also an integral component of treatment. The role for long course, external beam radiation therapy in pancreatic cancer, however, remains nebulous. This approach allows full, systemic doses of chemotherapy to be delivered without interruption and allows the expeditious delivery of high dose radiation therapy before definitive surgical management.

## Conclusions

In summary, we report our experience in a subset of patients with locally advanced and borderline resectable pancreatic cancer who were treated with neoadjuvant chemotherapy and SBRT followed by resection. We found that this approach is safe and is tolerated well. In our experience, 92% achieved an R0 resection and 41.7% of patients demonstrated either complete or extensive pathologic response to treatment. This is a promising area for further exploration in this disease site, and we currently have an ongoing phase II trial at our institution exploring this approach.

## Competing interests

The authors have no financial or personal relationships with other people or organizations that could inappropriately influence this work.

## Authors’ contribution

MSR and AMK were responsible for data collection. MSR and AMK were responsible for data analysis. MSR, DEH, REW, HJZ, NB, RB, AJM, AEQ, and SAB were responsible for concept design. MSR, DEH, REW, HJZ, NB, AMK, BL, RB, AJM, and SAB authors were responsible for manuscript writing and editing. All authors read and approved the final manuscript.
